# Supplementary material for: CaBLAM: a high-contrast bioluminescent Ca2+ indicator derived from an engineered Oplophorus gracilirostris luciferase
Source: Nat Methods. 2025 Dec 2;23(1):205–15. doi: 10.1038/s41592-025-02972-0 (PMC12791009; doi:10.1038/s41592-025-02972-0)
Supplement: Supplementary file 2 — Reporting Summary [file 41592_2025_2972_MOESM2_ESM.pdf]

nature portfolio

Corresponding author(s): Shaner, Nathan C.

Last updated by author(s): Oct 25, 2025

Reporting Summary

Nature Portfolio wishes to improve the reproducibility of the work that we publish. This form provides structure for consistency and transparency in reporting. For further information on Nature Portfolio policies, see our Editorial Policies and the Editorial Policy Checklist.

Statistics

For all statistical analyses, confirm that the following items are present in the figure legend, table legend, main text, or Methods section.

n/a	Confirmed	
☐	☒	The exact sample size (*n*) for each experimental group/condition, given as a discrete number and unit of measurement
☐	☒	A statement on whether measurements were taken from distinct samples or whether the same sample was measured repeatedly
☐	☒	The statistical test(s) used AND whether they are one- or two-sided<br>*Only common tests should be described solely by name; describe more complex techniques in the Methods section.*
☒	☐	A description of all covariates tested
☐	☒	A description of any assumptions or corrections, such as tests of normality and adjustment for multiple comparisons
☐	☒	A full description of the statistical parameters including central tendency (e.g. means) or other basic estimates (e.g. regression coefficient) AND variation (e.g. standard deviation) or associated estimates of uncertainty (e.g. confidence intervals)
☒	☐	For null hypothesis testing, the test statistic (e.g. *F*, *t*, *r*) with confidence intervals, effect sizes, degrees of freedom and *P* value noted<br>*Give P values as exact values whenever suitable.*
☒	☐	For Bayesian analysis, information on the choice of priors and Markov chain Monte Carlo settings
☒	☐	For hierarchical and complex designs, identification of the appropriate level for tests and full reporting of outcomes
☒	☐	Estimates of effect sizes (e.g. Cohen's *d*, Pearson's *r*), indicating how they were calculated

Our web collection on statistics for biologists contains articles on many of the points above.

Software and code

Policy information about availability of computer code

Data collection	Data acquisition used a combination of commercial and open-source software: NIS-Elements AR v5.41.01 (Nikon Instruments) for microscopy control and image capture, CLARIOstar v5.40 E4 (BMG LABTECH) for plate reader measurements, Andor Solis 64-bit v4.32.30000.0 (Oxford Instruments) for EMCCD data acquisition, Arduino IDE v2.3.2 for device control, and Bonsai v2.8.5 for synchronized behavioral and photometric recordings.
Data analysis	Data were analyzed using Fiji/ImageJ v1.54d–f (NIH), Python v3.9.22, and MATLAB R2024b, with additional open-source packages including SUPPORT (https://github.com/NICALab/SUPPORT, accessed June 4 2025) and Suite2p v0.14.0 (https://github.com/MouseLand/suite2p). Custom Python and MATLAB analysis scripts are available at https://github.com/Shaner-Lab/CaBLAM.

For manuscripts utilizing custom algorithms or software that are central to the research but not yet described in published literature, software must be made available to editors and reviewers. We strongly encourage code deposition in a community repository (e.g. GitHub). See the Nature Portfolio guidelines for submitting code & software for further information.

Data

Policy information about availability of data

All manuscripts must include a data availability statement. This statement should provide the following information, where applicable:

- Accession codes, unique identifiers, or web links for publicly available datasets
- A description of any restrictions on data availability
- For clinical datasets or third party data, please ensure that the statement adheres to our policy

Raw and processed data sets from experiments performed in this study are freely available via the Brown Digital Repository (https://doi.org/10.26300/7sg5-w257) and as input data for reproducing analysis with the custom code developed in this study (https://github.com/Shaner-Lab/CaBLAM). Bacterial and mammalian expression plasmids encoding SSLuc, GeNL_SS, and CaBLAM will be deposited for distribution by AddGene (pending); prior to availability from AddGene, plasmids will be shared with non-profit researchers upon request to NCS.

Human research participants

Policy information about studies involving human research participants and Sex and Gender in Research.

Reporting on sex and gender	N/A
Population characteristics	N/A
Recruitment	N/A
Ethics oversight	N/A

Note that full information on the approval of the study protocol must also be provided in the manuscript.

Field-specific reporting

Please select the one below that is the best fit for your research. If you are not sure, read the appropriate sections before making your selection.

☒ Life sciences ☐ Behavioural & social sciences ☐ Ecological, evolutionary & environmental sciences

For a reference copy of the document with all sections, see nature.com/documents/nr-reporting-summary-flat.pdf

Life sciences study design

All studies must disclose on these points even when the disclosure is negative.

Sample size	No formal power analyses were conducted. Sample sizes were chosen based on prior experience with similar imaging and biochemical experiments to ensure sufficient replication for reproducible estimates of variance and statistical testing. Replicate numbers matched established practice for genetically encoded indicator characterization, and observed effect sizes consistently exceeded within-group variability.
Data exclusions	No data were excluded from any analysis.
Replication	All key experiments were independently repeated with separate biological or technical replicates and reproduced similar results. In vitro, cell-based, and in vivo datasets were validated across multiple preparations or animals, and all replication attempts yielded consistent outcomes within expected biological variation.
Randomization	NDNF-Cre individual mice were assigned randomly to receive AAV encoding CaBLAM or GCaMP6s.
Blinding	For in vivo comparisons of CaBLAM and GCaMP6s, Investigators were blinded to the identity of the sensor during analysis. Blinding was not practical for other experiments or analysis.

Reporting for specific materials, systems and methods

We require information from authors about some types of materials, experimental systems and methods used in many studies. Here, indicate whether each material, system or method listed is relevant to your study. If you are not sure if a list item applies to your research, read the appropriate section before selecting a response.

Materials & experimental systems

n/a	Involved in the study
☒	☐ Antibodies
☐	☒ Eukaryotic cell lines
☒	☐ Palaeontology and archaeology
☐	☒ Animals and other organisms
☒	☐ Clinical data
☒	☐ Dual use research of concern

Methods

n/a	Involved in the study
☒	☐ ChIP-seq
☒	☐ Flow cytometry
☒	☐ MRI-based neuroimaging

Eukaryotic cell lines

Policy information about cell lines and Sex and Gender in Research

Cell line source(s)	HeLa and U2-OS cell lines were obtained from ATCC. N2a cells were a gift from Douglas Black.
Authentication	Cell lines were not authenticated in this study, but HeLa and U2-OS were grown directly from ATCC stocks, which have been validated by ATCC.
Mycoplasma contamination	All cell lines tested negative for mycoplasma.
Commonly misidentified lines (See ICLAC register)	No commonly misidentified cell lines were used in this study.

Animals and other research organisms

Policy information about studies involving animals; ARRIVE guidelines recommended for reporting animal research, and Sex and Gender in Research

Laboratory animals	Cortices were dissected out from one P2 Sprague-Dawley rat for neuron culture. Rat pups were housed with the dam in a vivarium on a 12-hour reversed light–dark cycle and had free access to food and water.<br><br>Seven NDNF-Cre mice (2 female/5 male; 23-35 weeks old on imaging day; JAX stock #030757) were used for in vivo imaging to selectively express CaBLAM or GCaMP6s in neuron-derived neurotrophic factor (NDNF) expressing cortical layer 1 interneurons. Three additional NDNF-Cre mice (0 female/3 male, each 14 weeks old on imaging day) were injected with a pan-neuronal CaBLAM to test peripheral luciferin delivery. Mice were housed in a vivarium on a reversed light-dark cycle and had free access to food and water.<br><br>15 larval zebrafish (Danio rerio), 1 day post fertilization, were used for imaging experiments, including 3 individuals from each of the following transgenic lines: Is(nefma), Is(nefma) (non-transgenic sibling controls), Tg(GLAST), Tg(hcrtr2), and Tg(elavl3). All larvae used were on the mitfa -/- background to remove pigment cells. Existing driver lines were Tg(-6.7Tru.Hcrtr2:GAL4-VP16) 19, called Tg(hcrtr2), stl601Tg20, called Is(nefma), and psi1Tg21, called Tg(elavl3). In addition, two new transgenic lines were generated for this study. Adult zebrafish were maintained at 28.5 ºC under a standard 14/10-hour light/dark cycle.
Wild animals	N/A
Reporting on sex	Aside from using both male and female mice, sex was not considered in the study design. Zebrafish sex was not determined for the individuals imaged in this study. Sex was not determined for the rat pup used to prepare cortical neurons.
Field-collected samples	N/A
Ethics oversight	All procedures involving rats were approved by the Institutional Animal Care and Use Committee of UC San Diego.<br><br>All procedures involving mice were conducted in accordance with the guidelines of the National Institute of Health and with approval of the Animal Care and Use Committee of Brown University.<br><br>All procedures involving larval zebrafish (Danio rerio) were approved by the Institutional Animal Care and Use Committee (IACUC) at New York University Grossman School of Medicine.

Note that full information on the approval of the study protocol must also be provided in the manuscript.
