# Supplementary material for: CaBLAM: a high-contrast bioluminescent Ca2+ indicator derived from an engineered Oplophorus gracilirostris luciferase
Source: Nat Methods. 2025 Dec 2;23(1):205–15. doi: 10.1038/s41592-025-02972-0 (PMC12791009; doi:10.1038/s41592-025-02972-0)
Supplement: Supplementary file 4 — Peptide sequences of SSLuc, related OLucs and intermediate clones from this work, and CaBLAM. [file 41592_2025_2972_MOESM4_ESM.pdf]

>SSLuc
MVFTLEDFVGDWEQIAAYNLDQVLEQGGVSSVLQTLAVSVTPIQRIVRSGENGLKIDIHVIIPYEGLSADQMA
HIEEVFKVVYPVDDHHFKVIMEYGTLVIDGVTPNMLNYFGRPYEGIAVFDGKKITVTGTLWNGNKIIDERLIT
PEGSMLFRVTINGVTGYRLLKKISN
>GeNL_SS (mNGd10C-GF-d5N_SSLuc)
MVSKGEEDNMASLPATHELHIFGSINGVDFDMVGQGTGNPNDGYEELNLKSTKGDLQFSPWILVPHIGYGFHQ
YLPYPDGMSPFQAAMVDGSGYQVHRTMQFEDGASLTVNYRYTYEGSHIKGEAQVKGTGFPADGPVMTNSLTAA
DWCRSKKTYPNDKTIISTFKWSYTTGNGKRYRSTARTTYTFAKPMAANYLKNQPMYVFRKTELKHSKTELNFK
EWQKAFTGFEDFVGDWEQIAAYNLDQVLEQGGVSSVLQTLAVSVTPIQRIVRSGENGLKIDIHVIIPYEGLSA
DQMAHIEEVFKVVYPVDDHHFKVIMEYGTLVIDGVTPNMLNYFGRPYEGIAVFDGKKITVTGTLWNGNKIIDE
RLITPEGSMLFRVTINGVTGYRLLKKISN
>CaBLAM
MVSKGEEDNMASLPATHELHIFGSINGVDFDMVGQGTGNPNDGYEELNLKSTKGDLQFSPWILVPHIGYGFHQ
YLPYPDGMSPFQAAMVDGSGYQVHRTMQFEDGASLTVNYRYTYEGSHIKGEAQVKGTGFPADGPVMTNSLTAA
DWCRSKKTYPNDKTIISTFKWSYTTGNGKRYRSTARTTYTFAKPMAANYLKNQPMYVFRKTELKHSKTELNFK
EWQKAFTGFEDFVGDWEQTAAYNLDQVLEQGGVSSVLQTLAVSVTPIQRIVRSGENGLKIDIHVIIPYEGLSA
DQMAHIEEVFKVVYPVDDHHFKVIMEYGTLVIDGVTPNMLNYFGRPYEGIAVFDGKKITVTGTLWNGNKIIDE
RLITPEGSMLFRVTINSASSDSSRRKWNKTGHAVRAIGRLSSGGSGGSGGSGGSGGSNLPDQLTEEQIAEFKE
EFSLFDKDGDGTITTKELGTVMRSLGQNPTEAELQDMINEVDADGDGTIDFPEFLTMMARKMKYRDTEEEIRE
AFGVFDKDGIGYISAAELRHVMTNLGEKLTDEEVDEMIREADIDGDGQVNYEEFVQMMTAKSPAVTGYRLLEE
ISN
>CaBLAM_294W
MVSKGEEDNMASLPATHELHIFGSINGVDFDMVGQGTGNPNDGYEELNLKSTKGDLQFSPWILVPHIGYGFHQ
YLPYPDGMSPFQAAMVDGSGYQVHRTMQFEDGASLTVNYRYTYEGSHIKGEAQVKGTGFPADGPVMTNSLTAA
DWCRSKKTYPNDKTIISTFKWSYTTGNGKRYRSTARTTYTFAKPMAANYLKNQPMYVFRKTELKHSKTELNFK
EWQKAFTGFEDFVGDWEQTAAYNLDQVLEQGGVSSVLQTLAVSVTPIQRIVRSGENGLKIDIHVIIPYEGLSA
DQMAHIEEVFKVVYPVDDHHFKVIMEYGTLVIDGVTPNMLNYFGRPYEGIAVFDGKKITVTGTLWNGNKIIDE
RLITPEGSMLFRVTINSASSDSSRRKWNKTGHAVRAIGRLSSGGSGGSGGSGGSGGSNLPDQLTEEQIAEFKE
EFSLFDKDGDGTITTKELGTVMRSLGQNPTEAELQDMINEVDADGDGTIDFPEFLTMMARKMKYRDTEEEIRE
AFGVFDKDGWGYISAAELRHVMTNLGEKLTDEEVDEMIREADIDGDGQVNYEEFVQMMTAKSPAVTGYRLLEE
ISN
>CaBLAM_332W
MVSKGEEDNMASLPATHELHIFGSINGVDFDMVGQGTGNPNDGYEELNLKSTKGDLQFSPWILVPHIGYGFHQ
YLPYPDGMSPFQAAMVDGSGYQVHRTMQFEDGASLTVNYRYTYEGSHIKGEAQVKGTGFPADGPVMTNSLTAA
DWCRSKKTYPNDKTIISTFKWSYTTGNGKRYRSTARTTYTFAKPMAANYLKNQPMYVFRKTELKHSKTELNFK
EWQKAFTGFEDFVGDWEQTAAYNLDQVLEQGGVSSVLQTLAVSVTPIQRIVRSGENGLKIDIHVIIPYEGLSA
DQMAHIEEVFKVVYPVDDHHFKVIMEYGTLVIDGVTPNMLNYFGRPYEGIAVFDGKKITVTGTLWNGNKIIDE
RLITPEGSMLFRVTINSASSDSSRRKWNKTGHAVRAIGRLSSGGSGGSGGSGGSGGSNLPDQLTEEQIAEFKE
EFSLFDKDGDGTITTKELGTVMRSLGQNPTEAELQDMINEVDADGDGTIDFPEFLTMMARKMKYRDTEEEIRE
AFGVFDKDGNGYISAAELRHVMTNLGEKLTDEEVDEMIREADIDGDGWVNYEEFVQMMTAKSPAVTGYRLLEE
ISN
>eKL9h
MVFTLEDFVGDWEQTAAYNLDQVLEQGGVSSVLQTLAVSVTPIQRIVRSGENGLKIDIHVIIPYEGLSADQMA
HIEEVFKVVYPVDDHHFKVIMEYGTLVIDGVTPNMLNYFGRPYEGIAVFDGKKITVTGTLWNGNKIIDERLIT
PDGSMLFRVTINGVSGWRLFEKISN
>eKAZ-L9
MVFTLADFVGDWQQTAGYNQDQVLEQGGLSSVFQTLGVSVTPIQKIVLSGENGLKIDIHVIIPYEGLSGDQMG
HIEMIFKVVYPVDDHHFKIIMEYGTLVIDGVTPNMIDYFGRPYPGIAVFDGKQITVTGTLWNGNKIIDERLIN
PDGSLLFRVTINGVTGWRLCENILA
>eKAZ-L6
MVFTLADFVGDWQQTAGYNQDQVLEQGGLSSLFQALGVSVTPIQKIVLSGENGLKIDIHVIIPYEGLSGFQMG
LIEMIFKVVYPVDDHHFKIIMEYGTLVIDGVTPNMIDYFGRPYPGIAVFDGKQITVTGTLWNGNKIIDERLIN
PDGSLLFRVTINGVTGWRLCENILA

```
>NanoBit::pep86
MVFTLEDFVGDWEQTAAYNLDQVLEQGGVSSLLQNLAVSVTPIQRIVRSGENALKIDIHVIIPYEGLSADQMA
QIEEVFKVVYPVDDHHFKVILPYGTLVIDGVTPNMLNYFGRPYEGIAVFDGKKITVTGTLWNGNKIIDERLIT
PDGSMLFRVTINGVSGWRLFKKIS
>NanoLuc
MVFTLEDFVGDWRQTAGYNLDQVLEQGGVSSLFQNLGVSVTPIQRIVLSGENGLKIDIHVIIPYEGLSGDQMG
QIEKIFKVVYPVDDHHFKVILHYGTLVIDGVTPNMIDYFGRPYEGIAVFDGKKITVTGTLWNGNKIIDERLIN
PDGSLLFRVTINGVTGWRLCERILA
>eKAZ
MVFTLADFVGDWQQTAGYNQDQVLEQGGLSSLFQALGVSVTPIQKIVLSGENGLKIDIHVIIPYEGLSGFQMG
LIEMIFKVVYPVDDHHFKIILHYGTLVIDGVTPNMIDYFGRPYPGIAVFDGKQITVTGTLWNGNKIIDERLIN
PDGSLLFRVTINGVTGWRLCENILA
>OLuc
MVFTLADFVGDWQQTAGYNQDQVLEQGGLSSLFQALGVSVTPIQKVVLSGENGLKADIHVIIPYEGLSGFQMG
LIEMIFKVVYPVDDHHFKIILHYGTLVIDGVTPNMIDYFGRPYPGIAVFDGKQITVTGTLWNGNKIYDERLIN
PDGSLLFRVTINGVTGWRLCENILA
```
